# Unraveling Complexity about Childhood Obesity and Nutritional Interventions: Modeling Interactions Among Psychological Factors

**DOI:** 10.1038/s41598-019-55260-1

**Published:** 2019-12-11

**Authors:** Keith Feldman, Gisela M. B. Solymos, Maria Paula de Albuquerque, Nitesh V. Chawla

**Affiliations:** 1 Department of Computer Science and Engineering, Interdisciplinary Center for Network Science and Applications (iCeNSA), University of Notre Dame, Notre Dame, IN 46556 USA; 2 Health Services and Outcomes Research, Children’s Mercy Kansas City, Kansas City, MO USA; 3 Department of Pediatrics, University of Missouri-Kansas City School of Medicine, Kansas City, MO USA; 4 Kellogg Institute for International Studies, University of Notre Dame, Notre Dame, IN USA; 5 Department of Physiology, Section Physiology of Nutrition, Federal University of São Paulo (UNIFESP), São Paulo, Brazil; 6 CREN, São Paulo, Brazil

**Keywords:** Risk factors, Computer science

## Abstract

As the global prevalence of childhood obesity continues to rise, researchers and clinicians have sought to develop more effective and personalized intervention techniques. In doing so, obesity interventions have expanded beyond the traditional context of nutrition to address several facets of a child’s life, including their psychological state. While the consideration of psychological features has significantly advanced the view of obesity as a holistic condition, attempts to associate such features with outcomes of treatment have been inconclusive. We posit that such uncertainty may arise from the univariate manner in which features are evaluated, focusing on a particular aspect such as loneliness or insecurity, but failing to account for the impact of co-occurring psychological characteristics. Moreover, co-occurrence of psychological characteristics (both child and parent/guardian) have not been studied from the perspective of their relationship with nutritional intervention outcomes. To that end, this work looks to broaden the prevailing view: laying the foundation for the existence of complex interactions among psychological features. In collaboration with a non-profit nutritional clinic in Brazil, this paper demonstrates and models these interactions and their associations with the outcomes of a nutritional intervention.

## Introduction

With associations to type-II diabetes, ischaemic stroke, heart disease, and various cancers, childhood obesity represents one of today’s most serious threats to children’s health and quality of life^1–6^. Childhood obesity is associated with a lifetime of health concerns, ranging from increased health care costs^4,7,8^ to placing additional stressors on family dynamics^9^. Despite global awareness of this issue, prevalence of overweight and obese children (ages 2–19) has risen 47.1% worldwide over the 33 years between 1980 and 2013^10^.

As a result, both practitioners and researchers have undertaken efforts to develop a wide collection of interventions targeting the growing population of at-risk children^11^. Traditionally, these interventions focused solely on nutritional aspects of a child’s life. However, recent literature has found the roots of obesity to be far more complex. This complexity is perhaps best captured in the 2010 work of Puder and Munsch, which notes, “Rather than a stable condition, childhood obesity represents a dynamic process, in which behavior, cognition and emotional regulation interact mutually with each other, with biological parameters, as well as with contextual factors, such as parental attitudes and familial eating, activity and nutritional patterns”^12^.

Driven by these findings, interventions are rapidly broadening the scope of factors they address. Among the more prominent, has been the inclusion of psychological characteristics, both from the child and his or her family^1,12–15^. As a result, emerging research studies have since provided evidence around the existence of associations between specific psychological characteristics and presence of obesity in children^1,14,16,17^. However, these studies have focused on univariate analysis of psychological characteristics with respect to childhood obesity^12,15,16,18^.

Consequently, when seeking insights beyond prevalence rates, studies evaluating associations to intervention and prevention efforts often result in conflicting and ambiguous results. Therefore, it remains unclear how these psychological relationships may drive the success of intervention strategies. Our work addresses exactly this uncertainty. We posit there is need to advance beyond associations to univariate characteristics and establish the importance of *interactions* between such characteristics as they pertain to intervention outcomes.

Our study focused on children (aged 0–10) and their families who received treatment at the Centre for Nutritional Recovery and Education (CREN), a large non-for-profit NGO in São Paulo, Brazil. CREN represents a unique study center offering patients a two-year interdisciplinary treatment program. As part of the program, healthcare professionals identify and treat comorbidities associated with a child’s obesity, evaluate the child’s body composition through bio-impedance and follow life-style habits with daily records of activity, sleep and screen time, and nutritionists provide detailed counseling on a family’s diet. Additionally, CREN offers group sessions where physical activities and nutritional workshops are provided for children and their guardians^1^. Finally, interwoven through each service is a registered psychologist who provides support to understand the elements underlying a child’s nutritional state^2^.

Together with nutritional evaluations, the structured psychological evaluations offer a foundation on which to study the complexities among co-occurring characteristics and their ties to childhood obesity treatment outcomes. Specifically, we consider two distinct analyses. The first aims to quantify how associations with an intervention outcome vary significantly when the expression of multiple psychological characteristics in a child and/or guardian is leveraged, compared to examining each characteristic alone. The second analysis involves building co-occurrence networks of psychological characteristics, and finding the networks structural and connectivity properties that differ significantly between intervention outcomes. This findings in this paper provide an opportunity for caseworkers, intervention planners, and researchers to develop more complete profiles of the children and families they serve, thereby improving nutritional outcomes.

## Results

The results of each of the primary analyses can be found in the respective subsections to follow, while a broader consideration around how such observations relate to complexity in the treatment of children at CREN is provided within the Discussion section.

We, first, highlight the different ways to measure nutritional outcomes that are based on anthropometric measures such as the change in body mass index for age (BMI/Age). However, at high degrees of obesity, these measures are often insufficient to represent changes in overall health. Thus, in order to capture also a more comprehensive measure of improvement, we derived a BMI thereshold for measuring improvement through an analysis of clinical tests; additional details about this measurement are included in the Methods section. Based on this BMI threshold, we divide the study population into two groups: Those with a significant improvement in BMI score (henceforth referred to as IMPV, n:128); and those who exhibited minimal or insignificant change (deemed MC, n:372).

### Baseline

**Figure 1:**
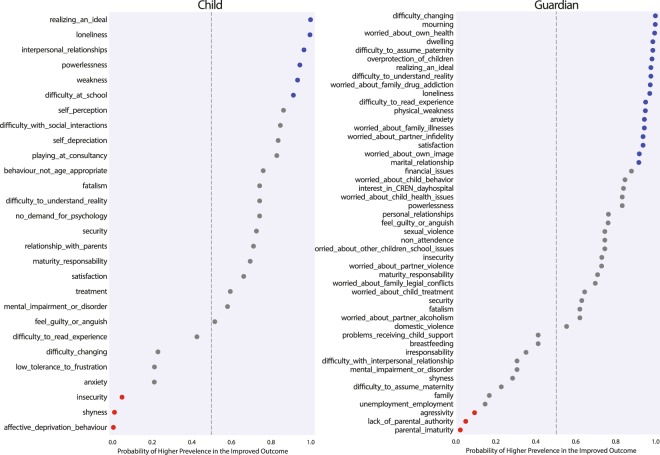
Baseline Tendencies: Probability of each characteristic to occur at a higher prevalence in the IMPV outcome. Colors: Blue nodes represent those at ≥90% probability of a higher prevalence in the IMPV group, while red indicates those with a ≥90% probability of occurring at a higher prevalence in the MC population — (≤10% probability for IMPV).

In order to better study the interactions amongst psychological characteristics and their association with childhood obesity, we first established the baseline tendency for each characteristic to occur within each of IMPV and MC groups. This was accomplished by generating distributions of occurrence prevalence for each psychological characteristic and computing the probability that it may occur at a higher prevalence within children of either the IMPV or MC group. A visual representation of these probabilities can be found in Figure 1. For additional details of this relationship, a comprehensive set of epidemiology-based statistics: odds ratio, absolute risk reduction, and population attributable risk percent, are provided in Supplementary Tables S1 and S2.

We observe several psychological characteristics with a probability ≥90% of occurring at a higher prevalence within one of outcome populations. For the IMPV population, these included elements of *loneliness*, *willingness to discus interpersonal relationships during the interview*, *difficulty changing*, *and concern about their own health*. While, the MC cohort exhibited increased occurrence probabilities for characteristics such as *guardian elements of immaturity*, *aggressiveness*, *lack of authority*, *and child affective deprivation behavior* (*in which a child must act out to receive attention*), *shyness*, *and insecurity*. It is important to note that because a psychological characteristic was observed to have a higher prevalence among the IMPV group, does not imply the characteristic is an inherently positive trait.

### Dyadic interactions

Our first analysis focused on interactions among the pair-wise (or dyadic) groupings of psychological characteristics, evaluating the prevalence of co-occurrences with respect to the baseline (univariate) tendencies of each element comprising the pair.

We first used an approach similar to estimating univariate tendencies among the outcome groups. Each dyad was considered as a unique entity and the probability of the dyad occurring at a higher prevalence in one of the outcome populations was computed. To better elucidate the influence of the interaction, only dyads where the baseline tendency of at least one characteristic exceeded 90% (for either IMPV or MC) were considered. For clarity, this characteristic will be referred to as the *seed variable*. Additional steps taken to reduce the possibility of spurious relations are documented in the Methods Section.

**Figure 2:**
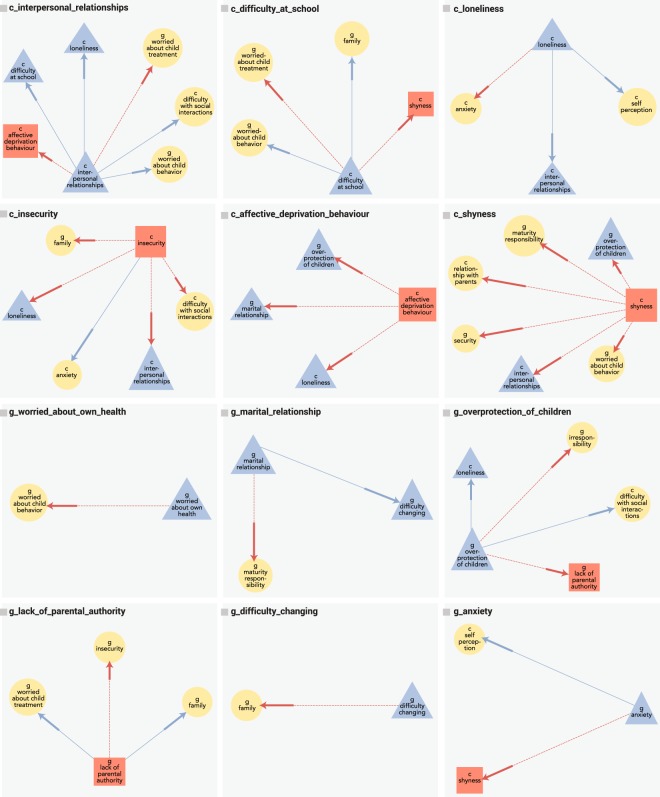
Dyadic Groupings: A visualization of the Stay-Swap relations at a minimum-probability of 90%. *Node Colors*: Blue nodes represent characteristics with a baseline above minimum-probability in the IMPV group, while red are those above minimum-probability in the MC outcome. Yellow nodes represent those not exceeding the minimum-probability in either outcome, yet were part of a dyad which did. *Edge Colors*: Blue edges connect characteristics with a stay relation, Red edges connect those with a swap relation.

In an effort to aid in comparisons between the dyadic and baseline probabilities, we designed a metric to convey a sense of direction for each interaction. This metric, deemed “Stay/Swap”, was defined as follows: If a dyad had a statistically higher prevalence in the *same* nutritional outcome as the seed variable, the interaction is designated as a “stay”. While dyads where the probability of occurrence was higher in the *opposite* outcome than the seed were deemed a “swap”. A visualization of all stay and swap relations can be seen in Figure 2. As with the baseline analysis, Supplementary Tables S3 and S4 present the epidemiological statistics assessing the prevalence of each dyad with respect to the IMPV group.

Evaluating dyadic interactions presented several interesting findings. For example, while the seed variable of *loneliness* was strongly associated with children in the IMPV population, the addition of *anxiety* to the initial assessment resulted in a *swap*: where the dyad becomes strongly associated with the MC population. In a similar fashion, children who speak about their interpersonal relationships with the psychologist are typically associated with IMPV outcomes; however, a swap is noted when they also exhibit affective deprivation behaviors.

These results present evidence to support the value of not only looking at psychological interactions within children or their guardians, but interactions *between* children and guardians as well. For example, at the baseline, children with shyness tend to occur at higher prevalence in the MC population, however when presented alongside highly mature guardians, guardians that are worried about/discuss the child’s behavior, or guardians who act in an overprotective manner, the outcome is more likely to be IMPV.

Drawing on the promising observations at the dyadic level, we performed an additional analysis to consider larger co-occurrence groupings: evaluating interactions among groupings of three characteristics, known as a triad. A discussion of the methods, results and primary findings can be found in Supplementary Material.

### Network effect

Our second analysis considers the more complex interactions captured in a “network effect” of the psychological characteristics. To do so, we constructed two networks (see Methods section) with each representing the co-occurrence patterns of psychological characteristics for children within the IMPV and MC outcome populations. Within a network, each characteristic was represented as a node, and edges were constructed and weighted by the proportion of children who exhibited the co-occurrence of both factors within the specific population. Once constructed, a robust set of 16 metrics were computed to provide insight into various elements of connectivity, associativity, and clustering amongst the psychological characteristics.

Across our 16 metrics (Table 1), we observed several highly probable differences between clustering measures (average neighbor degree, all pairs node connectivity, clustering coefficient) and connectivity measures (closeness centrality, triangles, node clique number). The results clearly indicate that interactions among psychological characteristics manifest themselves differently within each of the two nutritional outcomes. Using a Bayesian Estimation method (BEST)^19,20^, to obtain a high-density interval (HDI) at 90%, we estimate the probability of each network measure being truly different within each intervention outcome. In this case, positive ranges capture larger values within the IMPV, and conversely negative ranges indicate higher metrics for the MC population.

**Table 1:** Structural Metrics differentiating connectivity, associativity & clustering between the psychological characteristics of each outcome network.

Network Metric	Mean IMPV	Mean MC	HDI 5%	HDI 95%
*average_neighbor_degree*	31.70	35.81	−5.24	−2.88
*degree_centrality*	0.40	0.47	−0.14	0.00
*closeness_centrality*	66.56	187.34	−124.82	−118.01
*betweenness_centrality*	0.02	0.02	0.00	0.00
*edge_betweenness_centrality*	0.00	0.00	0.00	0.00
*number_of_cliques*	53.25	44.64	−12.20	18.36
*node_clique_number*	12.78	16.71	−5.22	−2.63
*triangles*	212.44	302.29	−149.86	−28.13
*clustering*	0.07	0.05	0.02	0.03
*all_pairs_node_connectivity*	15.46	18.84	−5.20	−1.43
*closeness_vitality*	186.07	177.29	−0.87	17.99
*square_clustering*	0.27	0.38	−0.14	−0.09
*edge_load*	82.20	80.94	0.79	1.77
*effective_size*	8.34	8.45	−2.16	2.19
*constraint*	0.13	0.11	0.00	0.01
*edge_weight*	0.02	0.02	0.00	0.01

## Discussion

This paper presents a strong case for the need to move beyond univariate psychological characteristics, and consider interactions among the psychological characteristics of both children and their parents/guardians. These interactions shed a more holistic light on the success of nutritional interventions to counter childhood obesity.

While the majority (59) of the 77 psychological characteristics were exhibited by children in both populations, a subset exhibited strong associations with one of the two nutritional outcomes. Such baseline associations offer a platform on which to study the novel associations between interactions of co-occurring psychological characteristics and the effectiveness of a nutritional intervention. Among the most prominent examples is child loneliness, which had a higher baseline probability of association with children in the IMPV population.

A potential explanation for such a connection comes as loneliness is often associated with the bullying by children that are overweight or obese. They are isolated by their friends, but literature has shown they also further isolate themselves^21,22^. As such, the association of loneliness among children with IMPV is driven mostly by the fact that treatment offered by CREN consists in part on the development of a companionship with the family, and aims to strengthen or increase a child’s social network breaking the vicious cycle of loneliness-eating-loneliness^23,24^.

However, when reviewing our interaction results, we find that when loneliness is paired with anxiety, it is more prevalent in the MC group. Literature suggests it is possible this combination captures a different profile of a child, representing any of the numerous generalized disorders whose underlying symptoms and causes stem outside the need to expand a child’s social circle^25^. Notably, recent works have highlighted important latent relations between these characteristics and underlying depression in children^26^. In both cases, the understanding of these profiles can improve future practice decisions; identifying population subgroups for behavioral or psychological interventions that accompany the nutritional intervention.

Additionally, interactions were found to occur in the reverse direction as well. For example, shyness was found to be more prevalent within the MC group at baseline. However, shyness, combined with the presence of a secure, responsible, or overprotective guardian, swaps the association to a higher probability of belonging to the IMPV group.

Such results highlight a key takeaway from our manuscript analysis, offering evidence around the value of strong family support in conjunction with an intervention. Where existing literature focuses mainly on how family stress may contribute to the development of child obesity^27,28^, there are studies and reviews that stress how parents contribute to the nutritional recovery of children, with the mother’s presence in the household^29^ and parent rules which diminish child consumption of sugars^30^. Other studies find elements to propose that the parents be the sole agents of change^31^.

Furthermore, our results support the notion that parents/guardians can contribute to their child’s nutritional status. A pattern that becomes more pronounced when looking to the triadic groupings (see Supplemental Materials). Across the numerous triads, we find the presence of strong family features such as the *guardian exhibiting concern for a child*’*s behavior and treatment* as an important element for successful intervention. We also find that extreme protectiveness for their child contributes to many interactions belonging to the IMPV group, whereas a lack of authority is steadily associated with MC.

On the other hand, curiously, certain attributes of the guardians psychological state, such as loneliness or insecurity, are connected to improvement in the child’s nutritional outcome, perhaps suggesting some family units could be identified as more willing to accept the advice and help from professionals at CREN.

Finally, looking at the network relationships of psychological characteristics and nutritional outcomes, we uncovered patterns of increased *edge load* and *clustering coefficient* occurring within the IMPV population. Together these metrics indicate characteristics in this group co-occur within a greater percentage of the population and alongside more shared elements than in the MC group respectively. This suggests, perhaps, a stronger underlying relationship amongst the characteristics expressed across the children who show substantial improvement.

In contrast, within the MC network, we find what are likely to be more distinct characteristic groupings across the population, represented in an elevated *average clique count* and *average neighbor degree*. The elevated *average clique count* indicates each characteristic was part of more isolated groupings (cliques), supporting the idea that characteristics presented by children in the MC population may not co-occur as strongly, perhaps suggesting multiple underlying psychological processes or conditions that would need to be addressed with supplemental treatment plans. Importantly, we note a low probability of difference between the total number of cliques within the two outcomes (similar mean values and high-density interval (HDI) that spans 0). In this case, the lack of significance is an important result, as it serves as a form of validation that the increased number of cliques for each characteristic observed in the MC group is not simply due to an increased number of possible cliques.

Interestingly, the *average neighbor degree* also indicates each characteristic was found to directly co-occur with other characteristics that have a high number of co-occurring edges. Such a measure is important, as it is unlikely that all possible co-occurrences were captured within the sample of 500 individuals. Thus, giving a notable elevation in this metric gives a potential area of future research, suggesting potential co-occurrences in higher dimensional groupings than were evaluated in this study.

We believe this manuscript takes an important first step in uncovering the psychological interactions associated with success of a nutritional intervention. It is our hope this study will provide a foundation on which future works can explore the complexity of psychological interactions on a broader scale, identifying how addressing co-occurring conditions can open up pathways for improved treatment of obese children. In particular, one of the most pertinent areas of future work falls to the study of temporal patterns, extending the associations found in this work and investigating how the addition, removal, or persistence of specific conditions throughout treatment influences the nutritional outcome of a child.

## Methods

### Data

The data utilized in this study were extracted directly from the electronic medical record of each child maintained by CREN. As CREN remained an active clinical entity during the course of this work, we obtained a snapshot of the child records on October 11^*th*^ 2017 to ensure a static dataset for analysis while the Centre continued day-to-day operations. The snapshot included approximately 7,800 children treated over a period of 8 years. However, this work focuses on a specific subset of 1,541 children who utilized psychological services available at CREN from 2013 to 2017. Broadly, two types of data were extracted for each child: psychological assessments (forming our psychological characteristics) and measures of nutritional status (forming the intervention outcome). Details of each are provided in the sections to follow. The study was approved by the Notre Dame institutional review board under protocol number 17-08-4032. All methods were performed in accordance with the relevant guidelines and regulations set forth by the Notre Dame IRB, CREN, and current Nature publications.

#### Psychological data

The psychological data were drawn from consultations with a registered psychologist, performed as part of a child’s interdisciplinary care at CREN. These data represent a set of characteristics surrounding the life experiences, treatment engagement, and perception of overall progress for both children and their guardians. Each consultation provides the opportunity for a psychologist to encode the expression of over 70 unique characteristics as they present within a child, and 89 exhibited by their guardians. Additional questions asked of guardians pertained to elements such as employment or marriage; not pertinent to a child. Together, these characteristics were selected from a list developed by CREN as a result of research studies^32^ and clinical working groups and are recorded directly into a child’s electronic health record.

Based on the scope of the present study, we incorporated 77 distinct psychological characteristics — 28 from children and 49 from guardians — for the modeling and analysis in this paper. However, it is important to note the variables themselves are not measures, but rather a record of psychological characteristics expressed by the child or guardian. As such, we do not compute any composite score, or rank based on the number or types of characteristics recorded. Finally, to prevent the introduction of bias from children who may be at varying stages of treatment, all data were drawn from a child’s first psychological assessment.

#### Nutritional data

Through several encounters with pediatricians and dietitians, a child’s treatment at CREN offers a reliable source of anthropometric data. As the work presented in this manuscript is focused on obesity, the subset of collected heights, weights, and ages, provides a fairly robust measure of nutritional status. While these measures are often computed as a ratio to obtain the Body Mass Index (BMI) for an individual, extensive work by the WHO has demonstrated that when computed for children there exists a need to further refine each metric with respect to age^33^. Thus, utilizing a set of Child Growth Standards developed by the WHO, measures of BMI for Age (BMI/A), and Height for Age (H/A) were computed for each child. However, it is well established that raw values of BMI/A and H/A are not sufficient to provide a comparative measure of a child’s nutritional status, and as such, the relative z-scores for BMI/A and H/A were computed against external reference distributions provided by the WHO^34–36^.

### Nutritional outcome

Nutritional recovery is a process that demands time, as it involves deep change of life habits of the children and their families. A systematic review of recovery obtained through various types of intervention found weight loss, ranging from 0.05 to 0.42 BMI z-score (standard deviation score of the body mass index) over a period of 12–24 months^37^. Yet, unfortunately, as many children treated at CREN exhibit such extreme levels of malnutrition, it is highly unlikely that many will “recover” to a level of healthy z-BMI/A over the course of their treatment^3^.

As such, nutritional outcomes at the Centre, are often measured by the relative improvement of child between their entrance and exit of treatment. While this relational measure offers valuable insight for the practitioners at CREN, the determination of a specific threshold of improvement that corresponds to a tangible improvement of health represents a challenging problem.

In an effort to obtain such an objective measure of change for use in this work, we look to a set of blood tests that provide a proxy: linking nutritional outcomes with known clinical biomarkers of health. Specifically, this work utilizes levels of Insulin, Triglycerides, and the Homeostatic Model Assessment of Insulin Resistance (HOMA-IR). These three exams were selected by the clinicians at CREN, and their link to nutritional outcomes has been established in a number of external research articles^38,39^.

#### Threshold definition

To begin we extracted the first and last recorded levels of each laboratory exam during each child’s treatment at CREN. Utilizing a subset of 72 children from our study population, each of whom also had all three blood tests recorded at least twice. We next sought to substantiate the relation between blood levels and nutritional outcomes established in literature. Utilizing a Mann-Whitney-U test (2-sided, 95% confidence) to compare the distribution of BMI/A z-score change for those children whose lab values showed improvements (a pre-post change in the direction towards a healthy range as defined by clinical standards), against those who show no change or a worsening (a pre-post change in the direction away a healthy range as defined by clinical standards) across all three blood tests. We define a significant reduction for those who improved their lab values at p < 0.001.

Noting such difference, the threshold for nutritional outcome improvement was set at the mean change in BMI z-score for those individuals with improved lab values across all three lab tests. While several alternative criteria were considered, requiring improvement in one or two of the various blood levels, literature suggests these lab values are typically “lag” indicators, i.e. they will move as a result of sustained improvement in nutritional status. Thus, requiring change towards healthy ranges in all 3 lab tests offered a robust proxy for improvement in overall nutritional status, as it also captures a decrease in the cardio-metabolic risks associated to obesity.

This process ultimately resulted in a threshold of 0.747 that, once applied to the study cohort^4^, resulted in two groups: children who substantially Improved (IMPV, n:128) in their nutritional status and those whose intervention resulted in minimal change (MC, n:372). Across these populations, age was well distributed — IMPV: (*μ:* 6.07 *years*, *σ*: 2.24), MC: (*μ*: 7.04 *years*, *σ*: 2.24). However, sex was found to be imbalanced, with a higher percentage of males in the improved group (64%, vs. 47%). While such differences are in line with known gender response differences to obesity interventions^40,41^, to guard against potential confounding of our results, we undertook an additional cohort control to remove any characteristics that had any statistical association to patient sex (details included in the Supplemental Material).

### Statistical methods

Each of the three primary analyses presented throughout this work employ Bayesian inference. The decision to utilize Bayesian inference over conventional hypothesis testing was driven by two primary considerations.

First, an extensive set of literature has emerged bringing to light a lack of reliability in the p-values required to determine what is considered “significant” with respect to various confidence levels of frequentist statistics^42,43^. In particular, work in the healthcare field has shown that such tests typically overstate the evidence against the null hypothesis^44^. Conversely, there exists prominent support for a Bayesian approach to perform comparisons between samples, particularly within the field of psychological analysis^45,46^.

Second, Bayesian statistics have been shown to provide an improved estimate of parameters in small populations. With respect to this study, although we have a fairly robust sample of 500 children, such considerations become valuable when evaluating dyadic and triadic interactions of infrequently expressed psychological characteristics^47^.

### Baseline

The establishment of baseline tendencies, performed independently for each of the psychological characteristics, was accomplished through two distinct procedures: (1) Epidemiological statistics (odds ratio, absolute risk reduction, and population attributable risk percent) were computed using a contingency table, breaking down a characteristic’s prevalence within each outcome; (2) as many characteristics occur within children of each outcome, a Bayesian estimation was performed to determine if the prevalence was truly different within each the IMVPV and MC outcomes. To do so, a Beta distribution was estimated for the prevalence of a characteristic within each outcome. Parameters *α* and *β* represented the occurrences of children who expressed or did not express the characteristic within the outcome respectively. Once established, a probability of their difference could be computed by obtaining the definite integral of the probability and cumulative density functions.

For consistency, all characteristics were evaluated with respect to the IMPV group, thus both very high and very low probabilities were of interest. For example, probability estimates of 90% indicate the characteristic has a 90% probability of occurring more in the IMPV outcome, while, by the conservation of probability, values of 10% represent 90% probability of occurring more in children with the MC outcome.

### Two characteristic groupings

As the number of possible dyadic groupings of 77 characteristics can become quite large, we took a number of steps to reduce the chances of spurious relations. First, we focused only on the subset of characteristics whose baseline tendency to occur within one of the two outcomes exceeded 90%. By using those characteristics with strong baseline tendencies, we can more rigorously evaluate how the addition of a characteristic may influence outcome prevalence probabilities.

For each psychological characteristic with a baseline probability above this threshold (referred to as the seed variables) the prevalence of co-occurring with each of the other 76 characteristics was then computed. In each case, the co-occurring dyad was considered a single entity. Then, as in the baseline analysis, the Bayesian comparison, and epidemiological statistics, were computed. Finally, should a dyad fail to reach a 90% probability of occurring within one of the two outcomes, or occurred five or less times between the two populations, the pair is disregarded to prevent overestimating interactions from extremely rare combinations.

### Network analysis

Each node in our network represents a unique psychological characteristic, while edges between characteristics indicate they have co-occurred within a child’s assessment (either from the child or the guardian). Additionally, each edge is weighted based on the percentage of children for whom the characteristic co-occur within the total population for the respective nutritional outcome.

Since, there are a subset of psychological characteristics that are expressed solely by children within only one nutritional outcome, inclusion of such elements could serve to bias analysis towards one outcome or another (IMPV vs MC). To mitigate this bias, all networks are constructed utilizing only the set of characteristics that are expressed in both IMPV and MC outcomes. While this may seem to be excluding relevant information, the understanding that it is highly unlikely any psychological characteristic exists solely within one outcome leads to the more reasonable assumption that the factor is infrequent, and not represented in the population sample evaluated in this study.

As a result, in order to include such a characteristic within the network it would be necessary to estimate its co-occurrence prevalence with all other characteristics. While such an estimation is possible it would often come with a strong bias. Rather, our goal was to evaluate the similarities and differences of how characteristics interact between the two outcome groups. Thus, by focusing the analyses within those shared by both groups, we can infer more accurate relationships between characteristic prevalence and nutritional outcomes.

Each metric is computed at the node-level for each of the networks. As an example, the clustering coefficient for each node was computed and stored, rather than a single average coefficient for the network. In this way, we can obtain a more comprehensive estimation of each metric value and variation across the characteristics.

To perform the comparison between each metric, we utilized the Bayesian Estimation Supersedes the t-test (BEST) method^19,20^. This method has been repeatably demonstrated as a strong estimate of continuous distributions. However, as published, the method relies on normal priors, yet in this work there is no guarantee the normality assumptions hold for these metric statistics. As a result, we instead turn to the gamma distributions to estimate priors, as structural measures do not extend to negative values. BEST estimations were sampled with JAGS, with a burn in of 1,000 iterations, and a total of 10,000 fitting iterations.

### Limitations

While every effort was taken to obtain a robust study cohort and perform rigorous evaluations for each analysis, there are of course limitations to the work presented in this manuscript.

The first centers around the socioeconomic status (SES) of the children and families treated at CREN. It is well known that SES factors are deeply tied to health and development of children. However, as children in both the IMPV and MC groups are drawn from the same population, an understanding of how SES may influence the associations between co-occurring psychological characteristics remains an open question. Although not a limitation of our study results, it is unclear how such findings would generalize to higher SES patient populations, where social support structures, self-perception, and coping mechanisms may differ. A question lending itself to the development of future multi-site studies.

The second limitation stems from the nature of data collection. As all data utilized in this study were collected through the natural course of care in the Centre, no child was evaluated by more than one psychologist. While all psychologists’ received extensive training at CREN and participated in case discussions with other practitioners, it is, of course, possible each had their own implicit biases in documenting specific characteristics. Unfortunately, without repeated cross-measurement of a child, it was not possible to capture inter-rater reliability metrics. However, we remain highly confident in their documented status, as the characteristics utilized in this work were selected from themes developed at the Centre for over 10 years and extensively discussed and tested by CREN’s psychologists.

The final limitation surrounds the demographic elements collected at CREN. Although we were able to assess the basic demographics of the children involved in the study, there exists a need to provide more complete information around race and ethnicity for future patients, particularly in regard to work around body composition. Yet, perhaps more importantly, is a limitation around demographic characteristics of the children’s guardians. Traditionally this information has not been collected at scale within CREN. However, the results of this study clearly indicate the importance of modeling the child-guardian dyad together and as such we believe it would be valuable to also study and control for the association of psychological characteristics within the guardians themselves. Moreover, although rare, challenging issues may also arise when children live in households with multiple families and caregivers, or travel between parents and/or grandparents for care throughout the year; perhaps requiring the modeling of multiple guardian characteristics. Through our current work at CREN, we are developing a more a more robust data collection procedure at a child’s enrollment to capture this type of information.

## Supplementary information

Supplementary Information

## Data Availability

The data that support the findings of this study are available on reasonable request from the corresponding author. The data are not publicly available due to privacy restrictions as they contain detailed aspects of health information.

## Footnotes

1 Guardian is broadly considered as the legal caregiver of the child.

2 A description of CREN’s enrollment and treatment program can be found in Supplementary Material.

3 CREN is a reference centre to treat obese children in the city of São Paulo. The 2 years treatment period was agreed upon in conjunction with the municipality based on the high demand for such a service in the city.

4 A detailed description of selection criteria is available in the supplementary material.
