# Supplementary material for: Unraveling Complexity about Childhood Obesity and Nutritional Interventions: Modeling Interactions Among Psychological Factors
Source: Sci Rep. 2019 Dec 11;9:18807. doi: 10.1038/s41598-019-55260-1 (PMC6906362; doi:10.1038/s41598-019-55260-1)
Supplement: Supplementary file 1 — Supplementary Information [file 41598_2019_55260_MOESM1_ESM.pdf]

Unraveling Complexity about Childhood Obesity and Nutritional Interventions: Modeling Interactions Among Psychological Factors

Keith Feldman[a,b,c], Gisela M. B. Solymos[a,d], Maria Paula Albuquerque[e], and Nitesh V. Chawla[a][1]

[a]Department of Computer Science and Engineering, Interdisciplinary Center for Network Science and Applications (iCeNSA), University of Notre Dame, IN 46556 USA
[b]Health Services and Outcomes Research Children's Mercy Kansas City, Kansas City, MO, USA
[c]Dept. of Pediatrics, University of Missouri-Kansas City School of Medicine, Kansas City, MO, USA
[d]Kellogg Institute for International Studies, University of Notre Dame
[e]Department of Physiology, Section Physiology of Nutrition, Federal University of São Paulo (UNIFESP), Brazil; CREN, São Paulo, Brazil

[1]Corresponding Author: nchawla@nd.edu

Treatment Program and Study Cohort

Treatment Program As noted in the main text, the data for this study were drawn from the Centre for Nutritional Recovery and Education (CREN), a large non-for-profit Brazilian NGO, founded in 1993. The Centre treats both undernourished and obese children and adolescents (0-19 years old) in day-hospitals, outpatient clinics and in poor communities across the cities of São Paulo and Maceio, in southeastern and northeastern of Brazil respectively.

Prior to treatment, children must be referred to the Centre's outpatient clinics for evaluation. Such a referral can occur in one of three ways: through professionals in the public health care system, through CREN's workers who conduct active searches in the city's slums and public schools, or by families contacting the Centre directly. In order to be admitted to the service, CREN's health staff evaluate each child to confirm the diagnosis of primary malnutrition.

At the onset of treatment, a child's first visit an evaluation by a nutritionist about the families' awareness of obesity as a health problem, and establishes therapeutic goals together with the family. Further, children's guardians are also invited to participate in a group session guided by a psychologist. Here they are introduced to the service as a whole and have the opportunity to share their uncertainties and experiences with other parents. Moving forward, CREN's ambulatory core treatment consists of one nutritional visit per month, a consultation with the physician every 3 months, and a consultation with the physical educator every six months. The nutritionist designs and follows-up on a strategy with the family to change their lifestyle habits in a manner that suits their budget, routines and needs. Finally, interwoven through each service, a psychologist provides support to the other professionals, either as part of a nutritional, clinical, or physical-educator consultation, or by conducting individual consultations under the professional's or the family's request.

CREN's methodology has been repeatedly demonstrated to be successful across a range of metrics, including promoting linear catch-up growth, appropriate gain in lean body mass and body mass composition (BMC), normal insulin production, and improvements to TG and HDL-C[1–4].

Study Cohort As the work presented in this manuscript investigates the complex nature of psychological characteristics, a considerable effort was made to control the study cohort for potential confounding effects. In particular accounting for elements of a child's developmental and physical state that may influence expression of various psychological factors. The following section will provide a detailed description of each selection criteria. For reference, all z-score thresholds utilized were drawn from the *Guidelines in the Management of Childhood Illness* provided by the WHO[5,6].

- Nutritional State at Enrollment:
  - First, as children can be admitted to CREN for treatment of either obesity or undernutrition, it is important to ensure the population contains a single physiological state, as each will result in specific treatment plans. This work aims to focus on obesity, and thus selected children admitted to CREN with a BMI/A z-score classified as obese (>2 standard deviations away for the average child in the WHO reference).
  - Building on this selection, we next ensure the child does not have an elevated BMI/A z-score due to a low height for age (H/A) z-score, which may be the result of stunted growth (another common condition at the Centre). To do so, all children whose H/A z-score classifies them at-risk, or stunted (H/A z-score <-1) were removed.
- Age
  - Moving on, the final selection step aims to account for the developmental effects of early adolescence. It has been well-established that the psychological characteristics early to late adolescent children are highly distinct[7,8]. With the age of puberty estimated at slightly above 10, we select all children aged 10 and younger for this study with the belief they provide a more consistent study population than those who may be a varying physiological development at the time of consultation[9,10].
- Sex
  - Finally, as the nutritional outcome populations were found to have differing proportions of male/female children, one last constraint was placed on the study cohort. Here, rather than restrict the patient population, we focused on the characteristics being evaluated, to ensure the assessment data on which this study would be built did not contain any implicit biases with respect to gender. For each of the 79 psychological characteristics, we evaluated the prevalence with a Fishers-Exact test at a confidence of 95% between the 255 male and 245 female children identified in the prior two cohort filtering steps. In the end, two characteristics demonstrated significant differences: one from the child assessment, (aggression) and one from their guardians (concern regarding the child at school). In both cases over-represented in the male population and were subsequently removed.

Upon completion, these filtering criteria resulted in a final study cohort of 500 children across 77 psychological characteristic. The initial psychological assessments were then matched with nutritional data for each child.

Supplementary Analysis 1: Triadic Variable Groupings

Supplementary Analysis 1: Triadic Variable Groupings

Methods: Similar to the Two Factor Grouping analysis in the main text, we first generated all combinations of three variables that were found to co-occur within the psychological assessments of each child and their families. However, due to the increased combinatorial space of possible variable configurations compared to the dyadic relationships, we take additional precautions to ensure not only that the relationships significantly are more likely to occur within one of the two outcomes, but also that any identified relations are representative of the population of children in each nutritional outcome and not an artifact of a few select children.

To do so, we utilize a repeated sampling technique known as bootstrap sampling. In this method (performed for each outcome independently), children are sampled randomly with replacement until the size of the original populations is reached. The set of triadic groupings were again extracted from the variables recorded in the psychological assessments of each child in the sample population. There exists substantial literature that discusses how by repeatedly performing this process (in this case 10,000 times) we can obtain a more accurate estimate of population statistics[11].

As we are estimating occurrences, we create a Beta distribution for each triad utilizing the number of bootstrap iterations in which the triad occurred as alpha, and the number in which it did not as beta. From each distribution, we then estimate credible interval (Bayesian version of a confidence interval) around the probability each triad truly exists within each outcome population. For consistency, we select those with a lower interval bound of over 90%.

Finally, utilizing the original populations of each nutritional outcome we computed the number of children in which each of the likely triads occurred. Utilizing another Beta distribution, we then computed the probability of each triad's occurrence being greater in the IMPV group compared to the MC group, ultimately selecting those with a 90% probability of occurring with a higher prevalence in one outcome.

Results: In total, a set of 7,273 unique triads were identified across both outcomes, 1,208 triads of whose occurrence was found to be highly probable within the population of each nutritional outcome through permutation testing. Of these, 142 triads were found to have a 90% probability of occurring with a higher prevalence in one outcome, forming the groupings utilized for the remainder of this analysis.

While the triads themselves represented the group of co-occurring variables whose prevalence differed significantly between nutritional outcomes, the interpretation of interactions is somewhat obfuscated due to the potential partial effects of each variable. Thus, to help better view such relations, all triads with exactly two matching variables were matched together, and the third (different) variable isolated. By evaluating how the variable (unmatched variable), influenced the association of a triad to each nutritional outcome, we were able to gain insight into how the addition of a third variable swapped the outcome with respect to the prevalence of the remaining matched groups. A list of all such groupings can be found in Supplementary Table S5.

Discussion: A close investigation of the nutritional outcome associated with these groupings highlights how an expansion to triadic groups can better define profiles of variables in relation to nutritional outcomes, as well as further elucidate relations found at the dyad level. For example, in the two variable interactions MC relations containing child's anxiety were found in combination with their insecurity or loneliness. Whereas, in the three-variable groupings, we see additional qualifications to these groups, where child's anxiety is found in triads with the guardian's maturity, responsibility, and concern about the child's behavior ultimately associated with a higher probability to improvement.

Such relations are interesting, as they lend credence to the hypothesis that variables may assume different meanings in the life of the child and their family, when as part of a larger profile including the set additional co-occurring variables presented by either entity. For example, where the dyads highlight the child's difficulties and the triads reveal the importance of the guardian's role for the child's nutritional outcome, or even as a resource for the child's life.

This pattern can be found across several other variables, such as the guardian's worry about the child's treatment. Where in the dyadic analysis this feature was connected to the child's interpersonal relationships, to the child's difficulty at school or to a lack of parental authority and was more likely associated to MC, in the triadic interactions it has different combinations that are associated to both outcomes.

Again, we note that some variables present such a strong relation to a specific outcome that regardless of those variables with which they co-occur the prevalence matches that of the univariate variable. For instance, triads that contain child affective deprivation behavior and all with lack of guardians' parental authority were all associated with MC outcome. Whereas those related to child's realization of an ideal (goal), discussion of interpersonal relationships with a psychologist, difficulty at school, and difficulty to change were all associated with the IMPV outcome.

References

1. Vieira, M. d. F. A., Ferraro, A. A., do Nascimento Souza, M. H., Fernandes, M. T. B. & Sawaya, A. L. Height and weight gains in a nutrition rehabilitation day-care service. *Public health nutrition* **13**, 1505–1510 (2010).
2. Alves, J. F., Britto, R. P., Ferreira, H. S., Sawaya, A. L. & Florêncio, T. M. Evolution of the biochemical profile of children treated or undergoing treatment for moderate or severe stunting: consequences of metabolic programming? *Jornal de Pediatr. (Versao em Portugues)* **90**, 356–362 (2014).
3. das Neves, J., Martins, P. A., Sesso, R. & Sawaya, A. L. Malnourished children treated in day-hospital or outpatient clinics exhibit linear catch-up and normal body composition. *The J. nutrition* **136**, 648–655 (2006).
4. Martins, V. J. *et al.* Long-lasting effects of undernutrition. *Int. journal environmental research public health* **8**, 1817–1846 (2011).
5. M., O. & T., L. Defining obesity risk status in the general childhood population: Which cut-offs should we use? *Int. J. Pediatr. Obes.* **5**, 458–460, DOI: 10.3109/17477161003615583.
6. World Health Organization and others. Guideline assessing and managing children at primary healthcare facilities to prevent overweight and obesity in the context of the double burden of malnutrition. (2017).
7. Dietz, W. H. Health consequences of obesity in youth: childhood predictors of adult disease. *Pediatrics* **101**, 518–525 (1998).
8. Sawaya, A. L. *et al.* Obesity and malnutrition in a shantytown population in the city of sao paulo, brazil. *Obesity* **3** (1995).
9. Luciano, A. P. *et al.* Median ages at stages of sexual maturity and excess weight in school children. *Reproductive health* **10**, 56 (2013).
10. Kelsey, M. M. & Zeitler, P. S. Insulin resistance of puberty. *Curr. diabetes reports* **16**, 64 (2016).
11. Efron, B. & Tibshirani, R. Bootstrap methods for standard errors, confidence intervals, and other measures of statistical accuracy. *Stat. science* 54–75 (1986).

Variable	ARR	Odds Ratio	OR CI	Population AR	prob_>impv	MC_T	MC_F	MC%	Impv_T	Impv_F	Impv_%
c_treatment	1.036519871	1.049562682	(0.65, 1.69)	0.825777202	0.595016021	84	288	0.225806452	30	98	0.234375
c_self_perception	1.199363733	1.28185907	(0.81, 2.02)	4.675066313	0.863504921	87	285	0.233870968	36	92	0.28125
c_interpersonal_relationships	1.419736667	1.638349515	(0.96, 2.79)	5.774297424	0.96671674	48	324	0.129032258	25	103	0.1953125
c_relationship_with_parents	1.114772727	1.159683794	(0.65, 2.08)	1.447821101	0.711961059	46	326	0.123655914	18	110	0.140625
c_difficulty_at_school	1.590163934	1.983606557	(0.69, 5.69)	1.739690722	0.913226372	9	363	0.024193548	6	122	0.046875
c_playing_at_consultancy	1.179908857	1.253844003	(0.77, 2.03)	3.573677582	0.829618264	73	299	0.196236559	30	98	0.234375
c_self_depreciation	1.960629921	2.921259843	(0.18, 47.05)	0.382781124	0.836391345	1	371	0.002688172	1	127	0.0078125
c_loneliness	1.941371681	2.771993753	(1.34, 5.73)	5.682425214	0.99702754	17	355	0.045698925	15	113	0.1171875
c_difficulty_to_understand_reality	nan	nan	(nan, nan)	nan	nan	0	372	0	0	128	0
c_mental_impairment_or_disorder	0.976190476	0.968253968	(0.19, 4.86)	-0.038109756	0.580986212	6	366	0.016129032	2	126	0.015625
c_difficulty_with_social_interactions	1.324929972	1.487394958	(0.65, 3.4)	1.724365751	0.847545549	18	354	0.048387097	9	119	0.0703125
c_insecurity	0.621468927	0.545762712	(0.27, 1.11)	-4.758522727	0.048781222	50	322	0.134408602	10	118	0.078125
c_behaviour_not_age_appropriate	1.309333333	1.464	(0.36, 5.94)	0.553716904	0.761035693	6	366	0.016129032	3	125	0.0234375
c_affective_deprivation_behaviour	0.351766513	0.283531409	(0.1, 0.81)	-5.758733624	0.005143877	38	334	0.102150538	4	124	0.03125
c_difficulty_changing	0.712134899	0.646711013	(0.24, 1.74)	-1.579016913	0.229920517	22	350	0.059139785	5	123	0.0390625
c_powerlessness	2.62962963	5.888888889	(0.53, 65.5)	0.968309859	0.945928312	1	371	0.002688172	2	126	0.015625
c_fatalism	nan	nan	(nan, nan)	nan	nan	0	372	0	0	128	0
c_realizing_an_ideal	3.075630252	9.302521008	(2.48, 34.92)	4.745133197	0.999869462	3	369	0.008064516	9	119	0.0703125
c_weakness	3.929133858	inf	(nan, inf)	0.58241483	0.934346447	0	372	0	1	127	0.0078125
c_low_tolerance_to_frustration	0.815423515	0.765084473	(0.42, 1.41)	-2.652616279	0.212363708	55	317	0.147849462	15	113	0.1171875
c_security	1.304461942	1.456692913	(0.13, 16.2)	0.182344064	0.726854377	2	370	0.005376344	1	127	0.0078125
c_anxiety	0.850607889	0.80717995	(0.49, 1.33)	-3.430269923	0.211613959	86	286	0.231182796	25	103	0.1953125
c_no_demand_for_psychology	1.306878307	1.46031746	(0.26, 8.07)	0.366902834	0.742777136	4	368	0.010752688	2	126	0.015625
c_feel_guilty_or_anguish	0.779527559	0.724409449	(0.08, 6.54)	-0.220959596	0.516799579	4	368	0.010752688	1	127	0.0078125
c_satisfaction	1.081952921	1.112912913	(0.61, 2.02)	1.005993151	0.662563112	45	327	0.120967742	17	111	0.1328125
c_maturity_responsability	1.154949785	1.219512195	(0.42, 3.53)	0.524068323	0.695402462	12	360	0.032258065	5	123	0.0390625
c_shyness	0.608233276	0.528099174	(0.3, 0.92)	-9.057741117	0.010658073	88	284	0.23655914	18	110	0.140625
c_difficulty_to_read_experience	0.648293963	0.577952756	(0.07, 4.99)	-0.423836032	0.426408556	5	367	0.01344086	1	127	0.0078125

Table S1: Epidemiological Statistics Comparing Prevalence of Child's Psychological Variables Between IMPV and MC Nutritional Outcomes.

Key: Absolute Risk Reduction (ARR), Odds Ratio (OR), Odds Ratio 95% Confidence Interval (OR CI), Population Attributable Risk Percent (Population AR), Probability of increased prevalence in IMPV outcome (prob_>impv), Children in MC Group with Risk (MC_T), Children in MC Group without Risk (MC_F), Prevalence in MC Group (MC%),Children in IMPV Group with Risk (Impv_T), Children in IMP Group without Risk (Impv_F), Prevalence in IMPV Group (Imp_%)

Note: As exhibiting a characteristic is a binary action, the number of children in each comparison group (n:IMPV or n:MC) can be determined by summing the total children who indicated the characteristic and those whose did not for each population respectively (IMPV_N: IMPV_T + IMPV_F or MC_N: MC_T + MC_F) . The total number of children (N) can subsequently be determined by summing all four categories.

Variable	ARR	Odds Ratio	OR CI	Population AR	prob_>impv	MC_T	MC_F	MC%	Impv_T	Impv_F	Impv_%
g_worried_about_partner_alcoholism	0.976377953	0.968503937	(0.1, 9.39)	-0.01890121	0.618050589	3	369	0.008064516	1	127	0.0078125
g_worried_about_partner_violence	1.304461942	1.456692913	(0.13, 16.2)	0.182344064	0.726854377	2	370	0.005376344	1	127	0.0078125
g_worried_about_partner_infidelity	3.929133858	inf	(nan, inf)	0.58241483	0.934346447	0	372	0	1	127	0.0078125
g_difficulty_to_assume_paternity	3.952380952	inf	(nan, inf)	1.167168675	0.983323997	0	372	0	2	126	0.015625
g_difficulty_to_assume_maternity	0	0	(0.0, nan)	-0.806451613	0.224911901	4	368	0.010752688	0	128	0
g_problems_receiving_child_support	0	0	(0.0, nan)	-0.401606426	0.409368323	2	370	0.005376344	0	128	0
g_worried_about_family_drug_addiction	2.271889401	3.967741935	(0.88, 17.97)	1.749492901	0.970447159	3	369	0.008064516	4	124	0.03125
g_worried_about_family_legial_conflicts	1.176	1.251428571	(0.32, 4.91)	0.350765306	0.695163932	7	365	0.018817204	3	125	0.0234375
g_worried_about_family_illnesses	1.807095344	2.479674797	(0.74, 8.27)	1.744631902	0.940874992	6	366	0.016129032	5	123	0.0390625
g_domestic_violence	0	0	(0.0, nan)	-0.200400802	0.55170933	1	371	0.002688172	0	128	0
g_sexual_violence			(nan, nan)		0.743027888	0	372	0	0	128	0
g_worried_about_child_treatment	1.057971014	1.079051383	(0.69, 1.69)	1.54109589	0.642049471	99	273	0.266129032	36	92	0.28125
g_worried_about_child_behavior	1.166666667	1.231481481	(0.82, 1.85)	6.25	0.843920801	144	228	0.387096774	56	72	0.4375
g_worried_about_other_children_school_issues			(nan, nan)		0.743027888	0	372	0	0	128	0
g_worried_about_child_health_issues	1.434017595	1.68202765	(0.48, 5.84)	0.945807771	0.830194334	7	365	0.018817204	4	124	0.03125
g_financial_issues	1.580645161	1.967741935	(0.55, 7.09)	1.147959184	0.876311275	6	366	0.016129032	4	124	0.03125
g_unemployment_employment	0.48015873	0.405895692	(0.09, 1.81)	-1.691632231	0.143963555	14	358	0.037634409	2	126	0.015625
g_worried_about_own_health	1.949832776	2.820512821	(1.21, 6.56)	4.186320755	0.992374348	12	360	0.032258065	11	117	0.0859375
g_worried_about_own_image	1.759856631	2.367741935	(0.63, 8.96)	1.349287169	0.915574076	5	367	0.01344086	4	124	0.03125
g_personal_relationships	1.309333333	1.464	(0.36, 5.94)	0.553716904	0.761035693	6	366	0.016129032	3	125	0.0234375
g_marital_relationship	1.328377096	1.488355681	(0.83, 2.68)	3.669400452	0.913347854	39	333	0.10483871	19	109	0.1484375
g_family	0.828274631	0.779468473	(0.48, 1.27)	-4.373346561	0.164767778	95	277	0.255376344	27	101	0.2109375
g_non_attendence	nan	nan	(nan, nan)	nan	nan	0	372	0	0	128	0
g_breastfeeding	0	0	(0.0, nan)	-0.401606426	0.409368323	2	370	0.005376344	0	128	0
g_mourning	2.862950058	7.520325203	(1.44, 39.26)	2.5418357	0.995416844	2	370	0.005376344	5	123	0.0390625
g_dwelling	3.952380952	inf	(nan, inf)	1.167168675	0.983323997	0	372	0	2	126	0.015625
g_interest_in_CREN_dayhospital	1.960629921	2.921259843	(0.18, 47.05)	0.382781124	0.836391345	1	371	0.002688172	1	127	0.0078125
g_overprotection_of_children	1.415770609	1.623655914	(1.02, 2.59)	8.030063291	0.979105927	70	302	0.188172043	35	93	0.2734375
g_loneliness	1.725090036	2.268907563	(0.93, 5.52)	2.955375783	0.967867135	12	360	0.032258065	9	119	0.0703125
g_difficulty_to_understand_reality	2	3	(0.95, 9.47)	2.34375	0.973552139	6	366	0.016129032	6	122	0.046875
g_mental_impairment_or_disorder	0	0	(0.0, nan)	-0.60362173	0.303539638	3	369	0.008064516	0	128	0
g_feel_guilty_or_anguish	1.171541502	1.243478261	(0.63, 2.46)	1.487116228	0.759405755	31	341	0.083333333	13	115	0.1015625
g_insecurity	1.116608595	1.162066182	(0.68, 1.97)	1.876495215	0.727858783	59	313	0.158602151	23	105	0.1796875
g_lack_of_parental_authority	0.586928105	0.510077519	(0.23, 1.12)	-4.398663697	0.046901281	43	329	0.115591398	8	120	0.0625
g_irresponsability	0.829039813	0.782414307	(0.31, 1.97)	-0.966631356	0.34867328	22	350	0.059139785	6	122	0.046875
g_difficulty_changing	1.980354267	2.89068323	(1.32, 6.33)	5.027748414	0.996121043	14	358	0.037634409	13	115	0.1015625
g_powerlessness	1.434017595	1.68202765	(0.48, 5.84)	0.945807771	0.830194334	7	365	0.018817204	4	124	0.03125
g_fatalism	0.976377953	0.968503937	(0.1, 9.39)	-0.01890121	0.618050589	3	369	0.008064516	1	127	0.0078125
g_realizing_an_ideal	2	3	(0.95, 9.47)	2.34375	0.973552139	6	366	0.016129032	6	122	0.046875
g_physical_weakness	2.62962963	5.888888889	(0.53, 65.5)	0.968309859	0.945928312	1	371	0.002688172	2	126	0.015625
g_difficulty_with_interpersonal_relationship	0	0	(0.0, nan)	-0.60362173	0.303539638	3	369	0.008064516	0	128	0
g_anxiety	1.364779874	1.547169811	(0.89, 2.7)	4.593894009	0.941130725	44	328	0.11827957	22	106	0.171875
g_parental_imaturity	0	0	(0.0, nan)	-2.459016393	0.019918849	12	360	0.032258065	0	128	0
g_security	1.066909091	1.092	(0.29, 4.18)	0.14698364	0.62764873	8	364	0.021505376	3	125	0.0234375
g_satisfaction	3.929133858	inf	(nan, inf)	0.58241483	0.934346447	0	372	0	1	127	0.0078125
g_maturity_responsability	1.090909091	1.125	(0.72, 1.76)	2.34375	0.706673139	96	276	0.258064516	36	92	0.28125
g_agressivity	0	0	(0.0, nan)	-1.419878296	0.0911096	7	365	0.018817204	0	128	0
g_shyness	0.484251969	0.410573678	(0.05, 3.37)	-0.832063008	0.281443825	7	365	0.018817204	1	127	0.0078125
g_difficulty_to_read_experience	1.983870968	2.967741935	(0.73, 12.04)	1.549796748	0.94706967	4	368	0.010752688	4	124	0.03125

Table S2: Epidemiological Statistics Comparing Prevalence of Guardian's Psychological Variables Between IMPV and MC Nutritional Outcomes.

Key: Absolute Risk Reduction (ARR), Odds Ratio (OR), Odds Ratio 95% Confidence Interval (OR CI), Population Attributable Risk Percent (Population AR), Probability of increased prevalence in IMPV outcome (prob_>impv), Children in MC Group with Risk (MC_T), Children in MC Group without Risk (MC_F), Prevalence in MC Group (MC%),Children in IMPV Group with Risk (Impv_T), Children in IMPV Group without Risk (Impv_F), Prevalence in IMPV Group (Impv_%)

Note: As exhibiting a characteristic is a binary action, the number of children in each comparison group (n:IMPV or n:MC) can be determined by summing the total children who indicated the characteristic and those whose did not for each population respectively (IMPV_N: IMPV_T + IMPV_F or MC_N: MC_T + MC_F) . The total number of children (N) can subsequently be determined by summing all four categories.

Seed Variable	OR1	Stay/Swap	Variable2	ARR	Odds Ratio	OR CI	Population AR	prob_>impv	MC_T	MC_F	MC%	Impv_T	Impv_F	Impv_%
c_interpersonal_relationships	0.96671674	Swap	g_worried_about_child_treatment	0.185271318	0.422705314	(0.15, 1.2)	-22.23255814	0.053586786	23	25	0.479166667	7	18	0.28
c_interpersonal_relationships	0.96671674	Same	c_loneliness	0.332051282	4.047058824	(1.16, 14.13)	17.26666667	0.987090533	5	43	0.104166667	8	17	0.32
c_interpersonal_relationships	0.96671674	Same	c_difficulty_with_social_interactions	0.353233831	4.380952381	(0.74, 25.83)	8.47761194	0.955010577	2	46	0.041666667	4	21	0.16
c_interpersonal_relationships	0.96671674	Swap	c_affective_deprivation_behaviour	0.296187683	0.158333333	(0.02, 1.32)	-13.03225806	0.032529519	10	38	0.208333333	1	24	0.04
c_interpersonal_relationships	0.96671674	Same	c_difficulty_at_school	0.253246753	2.857142857	(0.59, 13.93)	7.090909091	0.910113755	3	45	0.0625	4	21	0.16
c_interpersonal_relationships	0.96671674	Same	g_worried_about_child_behavior	0.184766214	2.289473684	(0.85, 6.15)	25.12820513	0.949363341	19	29	0.395833333	15	10	0.6
c_difficulty_at_school	0.913226372	Swap	g_worried_about_child_treatment	0.388888889	0.16	(0.01, 1.98)	-38.88888889	0.081756479	5	4	0.555555556	1	5	0.166666667
c_difficulty_at_school	0.913226372	Swap	c_shyness	0.75	0	(0.0, nan)	-87.5	0.00185109	7	2	0.777777778	0	6	0
c_difficulty_at_school	0.913226372	Same	g_family	0.444444444	7	(0.69, 70.75)	44.44444444	0.947758124	2	7	0.222222222	4	2	0.666666667
c_difficulty_at_school	0.913226372	Same	g_worried_about_child_behavior	0.482142857	10	(0.78, 128.78)	64.28571429	0.963595228	3	6	0.333333333	5	1	0.833333333
c_loneliness	0.99702754	Same	c_interpersonal_relationships	0.246963563	2.742857143	(0.64, 11.75)	21.40350877	0.909211523	5	12	0.294117647	8	7	0.533333333
c_loneliness	0.99702754	Swap	c_anxiety	0.458333333	0.102040816	(0.01, 0.97)	-24.44444444	0.014275121	7	10	0.411764706	1	14	0.066666667
c_loneliness	0.99702754	Same	c_self_perception	0.298701299	3.555555556	(0.73, 17.32)	41.81818182	0.939809128	9	8	0.529411765	12	3	0.8
c_insecurity	0.048781222	Swap	c_interpersonal_relationships	0.28	4.888888889	(1.06, 22.48)	28	0.981325702	6	44	0.12	4	6	0.4
c_insecurity	0.048781222	Swap	c_loneliness	0.37037037	6.714285714	(1.12, 40.07)	22.22222222	0.984779889	3	47	0.06	3	7	0.3
c_insecurity	0.048781222	Swap	c_difficulty_with_social_interactions	0.240384615	3.857142857	(0.75, 19.84)	19.23076923	0.955601512	5	45	0.1	3	7	0.3
c_insecurity	0.048781222	Swap	g_family	0.222222222	4	(0.97, 16.55)	33.33333333	0.974321954	10	40	0.2	5	5	0.5
c_insecurity	0.048781222	Same	c_anxiety	0.2	0	(0.0, nan)	-20	0.093701956	10	40	0.2	0	10	0
c_affective_deprivation_behaviour	0.005143877	Swap	c_loneliness	0.277777778	8.5	(0.93, 78.03)	41.66666667	0.977324292	4	34	0.105263158	2	2	0.5
c_affective_deprivation_behaviour	0.005143877	Swap	g_marital_relationship	0.1375	3.75	(0.45, 30.91)	34.375	0.909326635	8	30	0.210526316	2	2	0.5
c_affective_deprivation_behaviour	0.005143877	Swap	g_overprotection_of_children	0.162962963	6.5	(0.61, 69.14)	61.11111111	0.949053782	12	26	0.315789474	3	1	0.75
c_shyness	0.010658073	Swap	c_interpersonal_relationships	0.168055556	2.692307692	(0.8, 9.02)	14.9382716	0.952721198	11	77	0.125	5	13	0.277777778
c_shyness	0.010658073	Swap	g_security	0.35	5.666666667	(1.04, 30.77)	11.66666667	0.982492177	3	85	0.034090909	3	15	0.166666667
c_shyness	0.010658073	Swap	g_maturity_responsability	0.219409283	3.888888889	(1.35, 11.22)	32.91139241	0.994277751	18	70	0.204545455	9	9	0.5
c_shyness	0.010658073	Swap	c_relationship_with_parents	0.254166667	3.904761905	(0.98, 15.62)	14.12037037	0.976766304	6	82	0.068181818	4	14	0.222222222
c_shyness	0.010658073	Swap	g_overprotection_of_children	0.178694158	2.733333333	(0.62, 12.14)	8.934707904	0.927502843	6	82	0.068181818	3	15	0.166666667
c_shyness	0.010658073	Swap	g_worried_about_child_behavior	0.193441262	3.532608696	(1.24, 10.04)	35.46423135	0.991669253	23	65	0.261363636	10	8	0.555555556

Table S3: Epidemiological Statistics Comparing Prevalence of Child's Psychological Variable Dyads Between IMPV and MC Nutritional Outcomes.

Key: Odds Ratio of Seed Variable (OR1), Stay Swap Metric (Stay/Swap) -- All Remaining metrics are computed for the dyad -- Absolute Risk Reduction (ARR), Odds Ratio (OR), Odds Ratio 95% Confidence Interval (OR CI), Population Attributable Risk Percent (Population AR), Probability of increased prevalence in IMPV outcome (prob_>impv), Children in MC Group with Risk (MC_T), Children in MC Group without Risk (MC_F), Prevalence in MC Group (MC%),Children in IMPV Group with Risk (Impv_T), Children in IMPV Group without Risk (Impv_F), Prevalence in IMPV Group (Impv_%)

Note: As exhibiting a characteristic is a binary action, the number of children in each comparison group (n:IMPV or n:MC) can be determined by summing the total children who indicated the characteristic and those whose did not for each population respectively (IMPV_N: IMPV_T + IMPV_F or MC_N: MC_T + MC_F) . The total number of children (N) can subsequently be determined by summing all four categories.

Seed Variable	OR1	Stay/Swap	Variable2	ARR	Odds Ratio	OR CI	Population AR	prob_>impv	MC_T	MC_F	MC%	Impv_T	Impv_F	Impv_%
g_worried_about_own_health	0.992374348	Swap	g_worried_about_child_behavior	0.315384615	0.267857143	(0.05, 1.55)	-28.67132867	0.074895679	7	5	0.583333333	3	8	0.272727273
g_marital_relationship	0.913347854	Swap	g_maturity_responsability	0.292069632	0.161111111	(0.02, 1.37)	-16.90929451	0.037033305	10	29	0.256410256	1	18	0.052631579
g_marital_relationship	0.913347854	Same	g_difficulty_changing	0.217391304	2.538461538	(0.69, 9.32)	13.72997712	0.9230187	6	33	0.153846154	6	13	0.315789474
g_overprotection_of_children	0.979105927	Swap	g_lack_of_parental_authority	0.191675794	0.372759857	(0.12, 1.2)	-12.04819277	0.04964862	18	52	0.257142857	4	31	0.114285714
g_overprotection_of_children	0.979105927	Same	c_loneliness	0.188172043	2.206896552	(0.66, 7.43)	6.451612903	0.906309931	6	64	0.085714286	6	29	0.171428571
g_overprotection_of_children	0.979105927	Swap	g_irresponsability	0.353535354	0	(0.0, nan)	-6.060606061	0.051000618	6	64	0.085714286	0	35	0
g_overprotection_of_children	0.979105927	Same	c_difficulty_with_social_interactions	0.315721649	3.722222222	(0.83, 16.59)	7.216494845	0.962539484	3	67	0.042857143	5	30	0.142857143
g_lack_of_parental_authority	0.046901281	Same	g_worried_about_child_treatment	0.156565657	0.218487395	(0.02, 1.94)	-35.22727273	0.089678201	17	26	0.395348837	1	7	0.125
g_lack_of_parental_authority	0.046901281	Swap	g_insecurity	0.349206349	7.6	(1.43, 40.39)	39.28571429	0.992372702	5	38	0.11627907	4	4	0.5
g_lack_of_parental_authority	0.046901281	Same	g_family	0.175806452	0.180451128	(0.02, 1.6)	-43.9516129	0.056172114	19	24	0.441860465	1	7	0.125
g_difficulty_changing	0.996121043	Swap	g_family	0.261363636	0.342857143	(0.07, 1.68)	-32.16783217	0.098671717	10	4	0.714285714	6	7	0.461538462
g_anxiety	0.941130725	Swap	c_shyness	0.275	0.185185185	(0.02, 1.57)	-12.5	0.054482545	9	35	0.204545455	1	21	0.045454545
g_anxiety	0.941130725	Same	c_self_perception	0.254545455	2.925	(0.78, 10.97)	12.72727273	0.94707958	5	39	0.113636364	6	16	0.272727273

Table S4: Epidemiological Statistics Comparing Prevalence of Guardian's Psychological Variable Dyads Between IMPV and MC Nutritional Outcomes

Key: Odds Ratio of Seed Variable (OR1), Stay Swap Metric (Stay/Swap) -- All Remaining metrics are computed for the dyad -- Absolute Risk Reduction (ARR), Odds Ratio (OR), Odds Ratio 95% Confidence Interval (OR CI), Population Attributable Risk Percent (Population AR), Probability of increased prevalence in IMPV outcome (prob_>impv), Children in MC Group with Risk (MC_T), Children in MC Group without Risk (MC_F), Prevalence in MC Group (MC%),Children in IMPV Group with Risk (Impv_T), Children in IMPV Group without Risk (Impv_F), Prevalence in IMPV Group (Impv_%)

Note: As exhibiting a characteristic is a binary action, the number of children in each comparison group (n:IMPV or n:MC) can be determined by summing the total children who indicated the characteristic and those whose did not for each population respectively (IMPV_N: IMPV_T + IMPV_F or MC_N: MC_T + MC_F) . The total number of children (N) can subsequently be determined by summing all four categories.

Characteristic 1	Characteristic 2	Characteristic 3	Prob Difference
c_low_tolerance_to_frustration	g_worried_about_child_behavior	g_overprotection_of_children	93.85%
c_low_tolerance_to_frustration	g_worried_about_child_behavior	g_lack_of_parental_authority	4.97%
c_relationship_with_parents	g_worried_about_child_behavior	g_marital_relationship	97.04%
c_relationship_with_parents	g_worried_about_child_behavior	g_loneliness	92.43%
c_relationship_with_parents	g_worried_about_child_behavior	g_lack_of_parental_authority	9.11%
c_relationship_with_parents	g_worried_about_child_behavior	g_difficulty_changing	92.43%
c_relationship_with_parents	g_worried_about_child_behavior	g_irresponsability	95.96%
c_relationship_with_parents	g_worried_about_child_behavior	g_maturity_responsability	98.61%
c_self_perception	c_relationship_with_parents	g_lack_of_parental_authority	6.73%
c_self_perception	c_relationship_with_parents	c_realizing_an_ideal	99.58%
c_self_perception	c_relationship_with_parents	c_loneliness	99.39%
c_self_perception	c_treatment	g_anxiety	97.04%
c_self_perception	c_treatment	g_worried_about_child_treatment	9.46%
c_self_perception	c_treatment	g_loneliness	97.04%
c_self_perception	c_treatment	g_difficulty_changing	92.43%
c_self_perception	g_worried_about_child_treatment	g_maturity_responsability	8.82%
c_self_perception	g_worried_about_child_treatment	g_lack_of_parental_authority	6.73%
c_self_perception	g_worried_about_child_treatment	g_loneliness	92.43%
c_treatment	g_worried_about_child_treatment	g_realizing_an_ideal	99.58%
c_treatment	g_worried_about_child_treatment	g_lack_of_parental_authority	9.11%
c_treatment	g_worried_about_child_treatment	g_maturity_responsability	7.64%
c_treatment	g_worried_about_child_treatment	g_difficulty_changing	92.43%
g_family	c_low_tolerance_to_frustration	g_lack_of_parental_authority	3.67%
g_family	c_low_tolerance_to_frustration	g_difficulty_changing	92.43%
g_family	c_relationship_with_parents	g_lack_of_parental_authority	9.11%
g_family	c_relationship_with_parents	g_difficulty_changing	92.43%
g_family	c_relationship_with_parents	c_loneliness	91.56%
g_family	c_self_perception	g_lack_of_parental_authority	4.97%
g_family	c_self_perception	g_difficulty_changing	98.61%
g_family	c_self_perception	c_affective_deprivation_behaviour	2.48%
g_family	c_self_perception	c_loneliness	99.18%
g_family	c_treatment	g_difficulty_changing	95.96%
g_family	c_treatment	g_loneliness	92.43%
g_family	c_treatment	g_worried_about_child_treatment	7.54%
g_family	g_worried_about_child_treatment	g_feel_guilty_or_anguish	6.73%
g_family	g_worried_about_child_treatment	g_difficulty_changing	97.04%
g_family	g_worried_about_child_treatment	g_lack_of_parental_authority	3.67%
c_self_perception	c_interpersonal_relationships	g_maturity_responsability	95.61%
c_self_perception	c_loneliness	g_maturity_responsability	98.32%
c_self_perception	g_worried_about_child_treatment	g_maturity_responsability	8.82%
c_self_perception	c_affective_deprivation_behaviour	g_worried_about_child_treatment	8.82%
c_self_perception	c_realizing_an_ideal	g_worried_about_child_treatment	99.58%
c_self_perception	c_loneliness	g_worried_about_child_treatment	96.27%
c_self_perception	c_treatment	g_worried_about_child_treatment	9.46%
g_family	c_interpersonal_relationships	g_worried_about_child_behavior	91.32%
g_family	c_insecurity	g_worried_about_child_behavior	94.71%
g_family	c_affective_deprivation_behaviour	g_worried_about_child_behavior	4.16%
g_family	c_difficulty_at_school	g_worried_about_child_behavior	99.50%
g_family	c_loneliness	g_worried_about_child_behavior	97.04%
g_family	c_playing_at_consultancy	g_worried_about_child_treatment	91.32%
g_family	c_difficulty_with_social_interactions	g_worried_about_child_treatment	99.50%
g_family	c_affective_deprivation_behaviour	g_worried_about_child_treatment	2.70%
g_family	c_loneliness	g_worried_about_child_treatment	97.04%
g_family	c_treatment	g_worried_about_child_treatment	7.54%
g_worried_about_child_behavior	g_feel_guilty_or_anguish	g_maturity_responsability	6.73%
g_worried_about_child_behavior	g_anxiety	g_maturity_responsability	97.04%
g_worried_about_child_treatment	g_lack_of_parental_authority	g_overprotection_of_children	3.67%
g_worried_about_child_treatment	g_anxiety	g_overprotection_of_children	97.86%
g_worried_about_child_treatment	g_difficulty_changing	g_overprotection_of_children	99.58%
c_insecurity	c_self_perception	g_worried_about_child_treatment	6.73%
c_difficulty_with_social_interactions	c_self_perception	g_worried_about_child_treatment	98.32%
c_loneliness	c_treatment	g_worried_about_child_treatment	97.04%
c_self_perception	c_treatment	g_worried_about_child_treatment	9.46%
g_family	c_treatment	g_worried_about_child_treatment	7.54%
c_interpersonal_relationships	g_worried_about_child_behavior	g_maturity_responsability	99.42%
g_family	g_worried_about_child_behavior	g_maturity_responsability	7.64%
c_shyness	g_worried_about_child_behavior	g_maturity_responsability	91.32%
c_relationship_with_parents	g_worried_about_child_behavior	g_maturity_responsability	98.61%
c_anxiety	g_worried_about_child_behavior	g_maturity_responsability	94.09%

Table S5: Listing of three variable groupings which significantly differentiate children of each nutritional outcome

Key: Probability of differing prevalence between IMPV and MC outcome
